# Lifestyle is associated with atrial fibrillation development in patients with type 2 diabetes mellitus

**DOI:** 10.1038/s41598-021-84307-5

**Published:** 2021-02-25

**Authors:** Chan Soon Park, Kyung-Do Han, Eue-Keun Choi, Da Hye Kim, Hyun-Jung Lee, So-Ryoung Lee, Seil Oh

**Affiliations:** 1 Department of Internal Medicine, Seoul National University Hospital, 101 Daehak-ro, Jongno-gu, Seoul, 03080 Republic of Korea; 2 Graduate School of Medical Science and Engineering, Korea Advanced Institute of Science and Technology, Daejeon, Republic of Korea; 3 Department of Biostatistics, College of Medicine, The Catholic University of Korea, Seoul, Korea

**Keywords:** Cardiology, Epidemiology

## Abstract

We evaluated the impacts of lifestyle behaviors, namely smoking, alcohol consumption, and physical activity, on the development of new-onset AF in patients with DM. Using the Korean Nationwide database, we identified subjects diagnosed with type 2 DM and without previous history of AF between 2009 and 2012. Self-reported lifestyle behaviors were analyzed. Among 2,551,036 included subjects, AF was newly diagnosed in 73,988 patients (median follow-up 7.1 years). Both ex-smokers (hazard ratio [HR] 1.05, 95% confidence interval [CI] 1.02–1.07) and current smokers (HR 1.06, 95% CI 1.03–1.08) demonstrated a higher risk of AF than never smokers. Patients with moderate (15–29 g/day) (HR 1.12, 95% CI 1.09–1.15) and heavy (≥ 30 g/day) (HR 1.24, 95% CI 1.21–1.28) alcohol consumption exhibited an increased risk of AF, while subjects with mild alcohol consumption (< 15 g/day) (HR 1.01, 95% CI 0.99–1.03) had an AF risk similar to that of non-drinkers. Patients who engaged in moderate-to-vigorous physical activity showed a lower risk of AF (HR 0.93, 95% CI 0.91–0.94) than those who did not. This study suggests that smoking, alcohol consumption, and physical activity are associated with new-onset AF in patients with DM, and lifestyle management might reduce the risk of AF in this population.

## Introduction

Diabetes mellitus (DM) is a highly prevalent disease, and its global burden has increased tremendously during the past decades^1^. DM is one of the primary risk factors of cardiovascular diseases and is associated with an increased risk of cardiovascular mortality^2,3^. For this reason, various attempts have been made to enhance outcomes in patients with DM, and the importance of preventing and managing cardiovascular complications has been emphasized in clinical guidelines^4^. Indeed, the US Food and Drug Administration made a guide to evaluate the cardiovascular effect in novel anti-diabetic therapies, and several studies demonstrated that some anti-diabetic drugs exert cardiovascular benefit^5–7^. The major cardiovascular complication assessed in these studies was the risk of atherosclerotic cardiovascular disease (ASCVD), which was defined as coronary artery disease, cerebrovascular disease, and peripheral arterial disease.

Atrial fibrillation (AF) is the most common arrhythmia seen in clinical practice and imposes a substantial burden on society^8–10^. Patients with AF are known to have a higher risk of stroke, heart failure, and mortality^11^. Therefore, there is a demand for better risk stratification and tailored treatment for AF to further improve clinical outcomes in these patients. Hypertension, DM, and obesity are well-known risk factors of AF, and pre-hypertension, impaired fasting glucose, and abdominal obesity are known to be related to AF development^12^. A previous meta-analysis demonstrated that DM is associated with an increased risk of new-onset AF^13^, but few studies have investigated independent risk factors of AF in patients with DM^14,15^. Furthermore, there is a paucity of information regarding the association between DM and new-onset AF in current clinical guidelines^4,16^.

Though many drugs have shown significant efficacy in controlling hyperglycemia, lifestyle management still serves a pivotal role in diabetes care^17^. Smoking, alcohol consumption, and physical activity are considered traditional and important components of lifestyle management^17^. Previous studies have proved that these factors are associated with cardiovascular complications related to ASCVD in patients with DM^18–20^. However, few studies have investigated whether these factors are also related to AF.

In this study, we aimed to investigate the prognostic value of lifestyle behaviors including smoking, alcohol consumption, and physical activity in predicting new-onset AF among patients with DM. To explore this comprehensively, we analyzed nationwide epidemiologic data from the Korean National Health Insurance Service (NHIS) database.

## Results

### Clinical characteristics of the study population

Among 2,551,036 DM patients without a previous diagnosis of AF, 73,988 (2.9%) were newly diagnosed with AF during the median follow-up period of 7.1 years. The median follow-up of patients with AF was 3.8 years, while that of those without AF was 7.3 years. Table 1 presents the clinical characteristics of the study population and Table 2 presents the characteristics of patients according to new-onset AF during follow-up. In brief, the incidence of AF during follow-up was 4.09 per 1000 PY. Patients with AF were older, and a higher proportion of this population had a history of hypertension, hyperlipidemia, chronic kidney disease, or anti-diabetic treatment. In regards to lifestyle behaviors, patients with AF exhibited a lower prevalence of smoking and drinking. On the contrary, a higher proportion of patients without AF engaged in MVPA.

**Table 1:** Baseline characteristics of overall subjects.

	Total population (n = 2,551,036)
**Demographics**	
Age (years)	57.7 ± 11.9
< 50	634,286 (24.9)
50–59	754.438 (29.6)
60–69	683,925 (26.8)
≥ 70	478,387 (18.8)
Sex	
Male	1,527,141 (59.9)
Female	1,023,895 (40.1)
Low-income level	682,550 (26.8)
**Medical history**	
Hypertension	1,450,253 (56.9)
Hyperlipidemia	1,085,129 (42.5)
Chronic kidney disease	281,683 (11.0)
Pharmacologic therapy for diabetes	
Insulin	207,123 (8.1)
Oral antidiabetics	1,519,139 (59.6)
**Physical exam**	
BMI (kg/m^2^)	
< 18.5	40,338 (1.6)
18.5–22.9	640,812 (25.1)
23–24.9	642,830 (25.2)
≥ 25	1,227,056 (48.1)
Systolic blood pressure (mmHg)	129.1 ± 15.7
Diastolic blood pressure (mmHg)	79.1 ± 10.1
**Laboratory findings**	
Total cholesterol (mg/dL)	197.0 ± 42.0
HDL-cholesterol (mg/dL)	51.4 ± 16.8
LDL-cholesterol (mg/dL)	111.6 ± 37.7
Glomerular filtration rate (mL/min/1.73 m^2^)	85.2 ± 35.6
Fasting glucose (mg/dL)	14.1 ± 43.6
**Lifestyle**	
Smoking	
Non	1,427,015 (55.9)
Ex	467,444 (18.3)
Current	656,577 (25.7)
Alcohol consumption	
Non	1,462,406 (57.3)
Mild (< 15 g/day)	546,618 (21.4)
Moderate (15–29 g/day)	286,117 (11.2)
Heavy (≥ 30 g/day)	255,895 (10.0)
Exercise	
Moderate-to-vigorous physical activity^a^	
No	1,332,830 (52.3)
Yes	1,218,206 (47.8)

*AF* atrial fibrillation, *BMI* body mass index, *DM* diabetes mellitus, *HDL* high-density lipoprotein, *LDL* low-density lipoprotein.

^a^Moderate intensity at least 30 min/day or vigorous-intensity at least 20 min/day.

**Table 2:** Baseline characteristics of DM patients according to new-onset AF during the follow-up.

	No new-onset AF (n = 2,477,048)	New-onset AF (n = 73,998)	P value
**Demographics**			
Age (years)	57.5 ± 11.9	65.6 ± 10.3	< 0.001
< 50	629,214 (25.4)	5,072 (6.9)	< 0.001
50–59	740,221 (29.9)	14,217 (19.2)	
60–69	659,278 (26.6)	24,647 (33.3)	
≥ 70	448,335 (18.1)	30,052 (40.6)	
Sex			< 0.647
Male	1,482,789 (59.9)	44,352 (59.9)	
Female	994,259 (40.1)	29,636 (40.1)	
Low-income level	663,009 (26.8)	16,621 (22.5)	< 0.001
**Medical history**			
Hypertension	1,396,079 (56.4)	54,174 (73.2)	< 0.001
Hyperlipidemia	1,053,295 (42.5)	31,834 (43.0)	< 0.006
Chronic kidney disease	266,663 (10.8)	15,020 (20.3)	< 0.001
Pharmacologic therapy for diabetes			
Insulin	197,338 (8.0)	9,785 (13.2)	< 0.001
Oral antidiabetics	1,467,936 (59.3)	51,203 (69.2)	< 0.001
**Physical exam**			
BMI (kg/m^2^)			< 0.001
< 18.5	38,931 (1.6)	1,407 (1.9)	
18.5–22.9	622,420 (25.1)	18,392 (24.9)	
23–24.9	625,004 (25.2)	17,826 (24.1)	
≥ 25	1,190,693 (48.1)	36,363 (49.2)	
Systolic blood pressure (mmHg)	129.0 ± 15.6	131.2 ± 16.7	< 0.001
Diastolic blood pressure (mmHg)	79.1 ± 10.1	79.1 ± 10.6	0.705
**Laboratory findings**			
Total cholesterol (mg/dL)	197.2 ± 42.0	190.5 ± 41.7	< 0.001
HDL-cholesterol (mg/dL)	51.4 ± 16.8	51.1 ± 18.4	< 0.001
LDL-cholesterol (mg/dL)	111.8 ± 37.7	107.9 ± 37.2	< 0.001
Glomerular filtration rate (mL/min/1.73 m^2^)	85.4 ± 35.6	78.9 ± 36.2	< 0.001
Fasting glucose (mg/dL)	144.2 ± 43.6	140.3 ± 44.5	< 0.001
**Lifestyle**			
Smoking			< 0.001
Non	1,382,957 (55.8)	44,058 (59.6)	
Ex	452,530 (18.3)	14,914 (20.2)	
Current	641,561 (25.9)	15,016 (20.3)	
Alcohol consumption			
Non	1,415,519 (55.8)	46,887 (63.4)	
Mild (< 15 g/day)	533,609 (21.5)	13,009 (17.6)	
Moderate (15–29 g/day)	279,172 (11.3)	6,945 (9.4)	
Heavy (≥ 30 g/day)	248,748 (10.0)	7,147 (9.7)	
Exercise			
Moderate-to-vigorous physical activity^a^			< 0.001
No	1,289,397 (52.1)	43,433 (58.7)	
Yes	1,187,651 (48.0)	30,555 (41.3)	

*AF* atrial fibrillation, *BMI* body mass index, *DM* diabetes mellitus, *HDL* high-density lipoprotein, *LDL* low-density lipoprotein.

^a^Moderate intensity at least 30 min/day or vigorous-intensity at least 20 min/day.

### Clinical outcomes according to smoking, alcohol consumption, and physical activity

**Figure 1:**
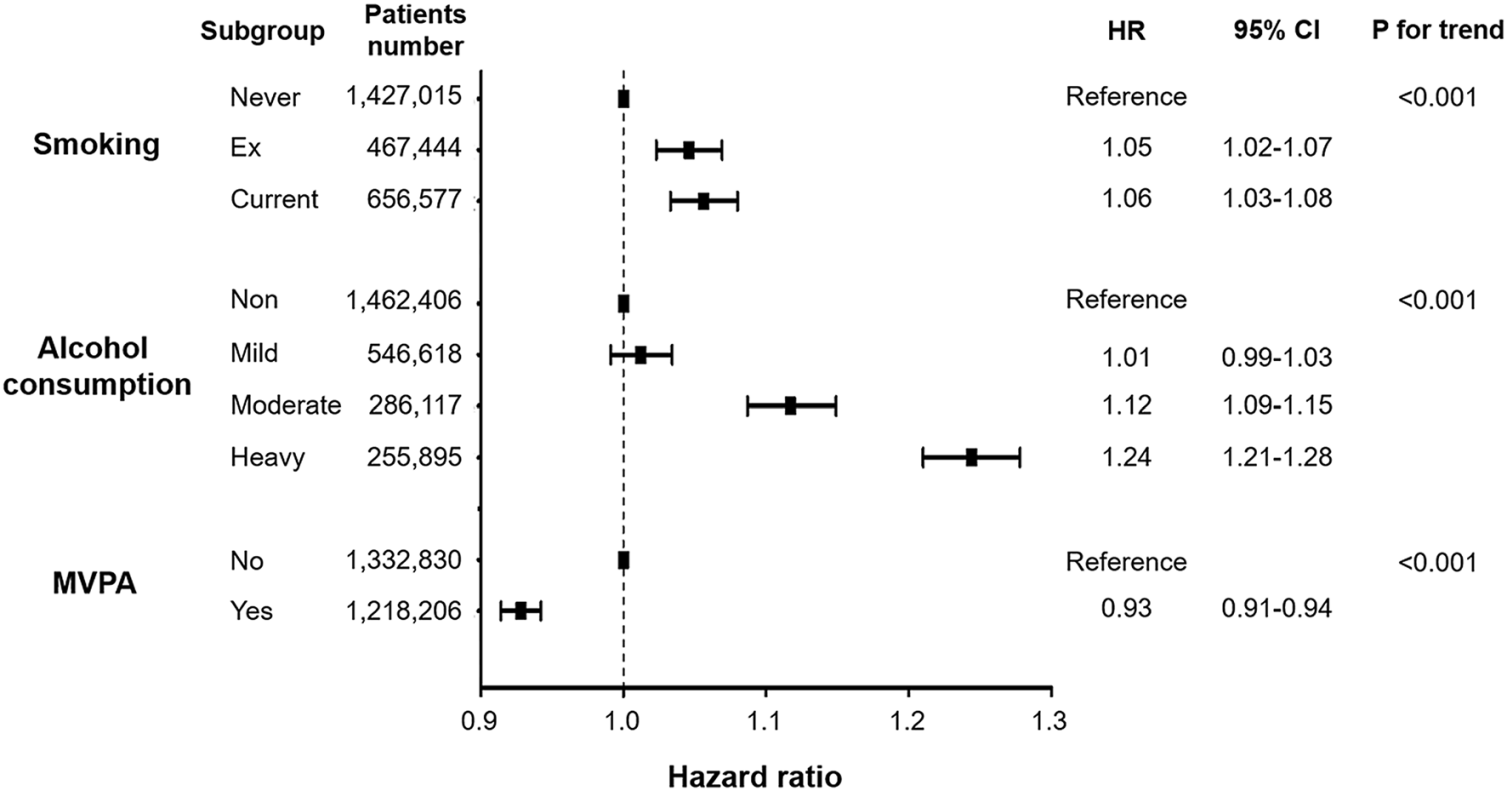
Clinical outcomes according to smoking, alcohol consumption, and physical activity. New-onset AF risk according to lifestyle behaviors in patients with DM. Data regarding lifestyle behaviors were collected during health check-ups as a self-reported questionnaire. *AF* atrial fibrillation, *CI* confidence interval, *DM* diabetes mellitus, *HR* hazard ratio, *MVPA* moderate-to-vigorous physical activity.

The associations between lifestyle behaviors and new-onset AF are demonstrated in Fig. 1 and Supplementary Table 1. With respect to tobacco smoking, the IRs of AF in never smokers, ex-smokers, and current smokers were 4.33, 4.53, and 3.26 per 1,000 PY, respectively. In the multivariate analyses, however, both ex-smokers (HR 1.05, 95% CI 1.02–1.07; model 3) and current smokers (HR 1.06, 95% CI 1.03–1.08) had a higher risk of AF than never smokers. The cumulative dose of smoking was stratified by the number of pack-years, and AF risk exhibited a positive correlation with cumulative smoking dose (Supplementary Fig. 1 and Supplementary Table 2).

With regards to alcohol consumption, the IRs of AF in non-, mild, moderate, and heavy drinkers were 4.50, 3.38, 3.45, and 3.97 per 1000 PY, respectively. After adjusting for covariates using model 3, mild drinkers (HR 1.01, 95% CI 0.99–1.03) had an AF risk similar to that of non-drinkers. However, moderate (HR 1.12, 95% CI 1.09–1.15) and heavy drinkers (HR 1.24, 95% CI 1.21–1.28) had a substantially higher risk of AF than non-drinkers. Subgroup analyses according to average daily alcohol consumption and weekly drinking frequency are presented in Supplementary Fig. 2 and Supplementary Table 3. A J-curve association was identified between alcohol consumption and new-onset AF.

With respect to physical activity, the IRs of AF in patients who engaged in MVPA and those who did not were 4.61 and 3.53 per 1000 PY, respectively, and patients who undertook MVPA exhibited a lower risk of AF in the multivariate analyses (HR 0.93, 95% CI 0.91–0.94; model 3). The individual associations between vigorous- and moderate-intensity physical activity and AF risk were also analyzed (Supplementary Fig. 3 and Supplementary Table 4).

### Subgroup analyses according to sex

**Figure 2:**
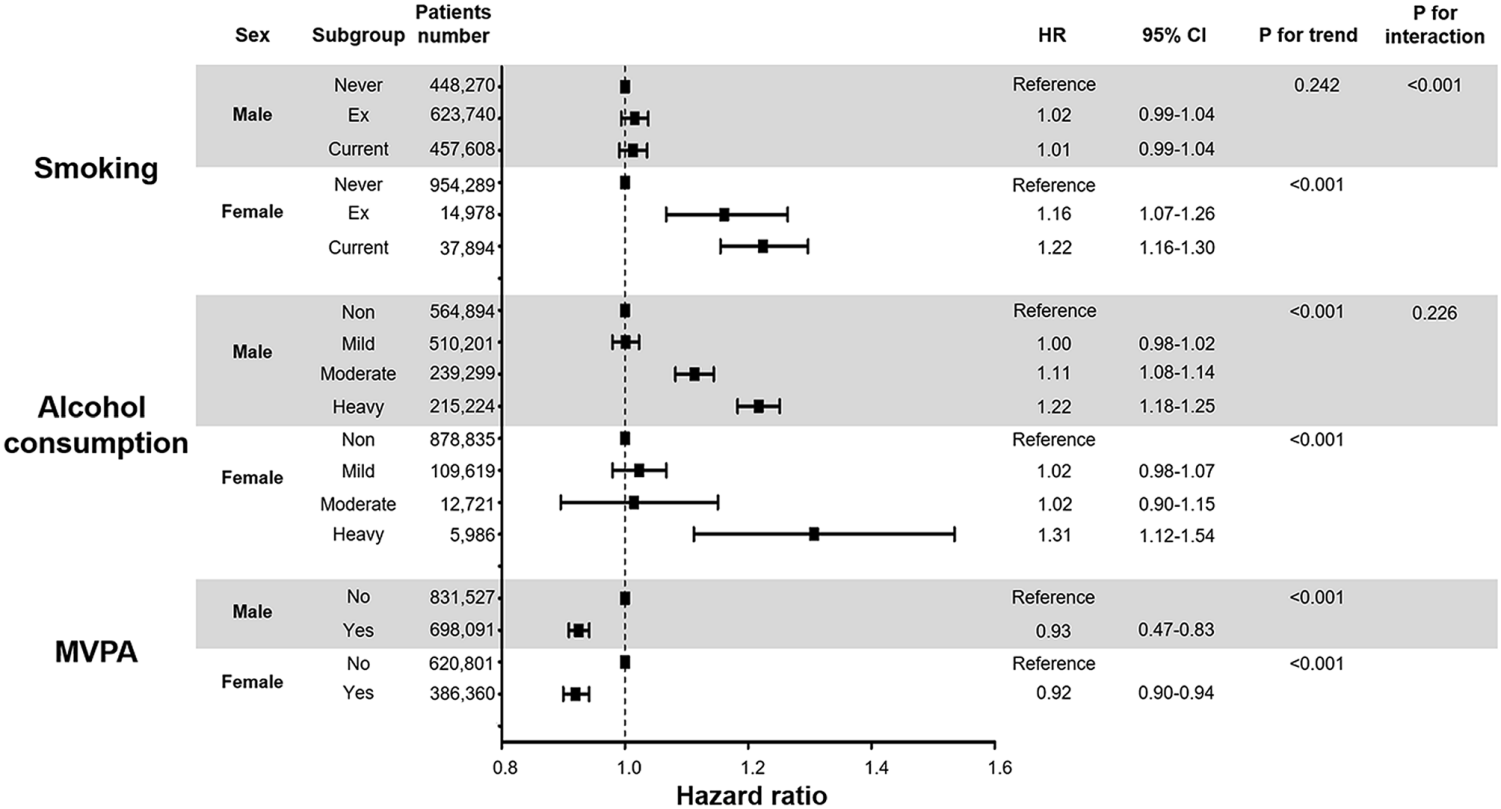
Relationship between lifestyle behaviors and new-onset AF according to sex. Subgroup analyses were performed to investigate the associations between smoking (**A**), alcohol consumption (**B**), and physical activity (**C**) and new-onset AF according to sex. Data regarding lifestyle behaviors were collected during health check-ups as a self-reported questionnaire. *AF* atrial fibrillation.

**Figure 3:**
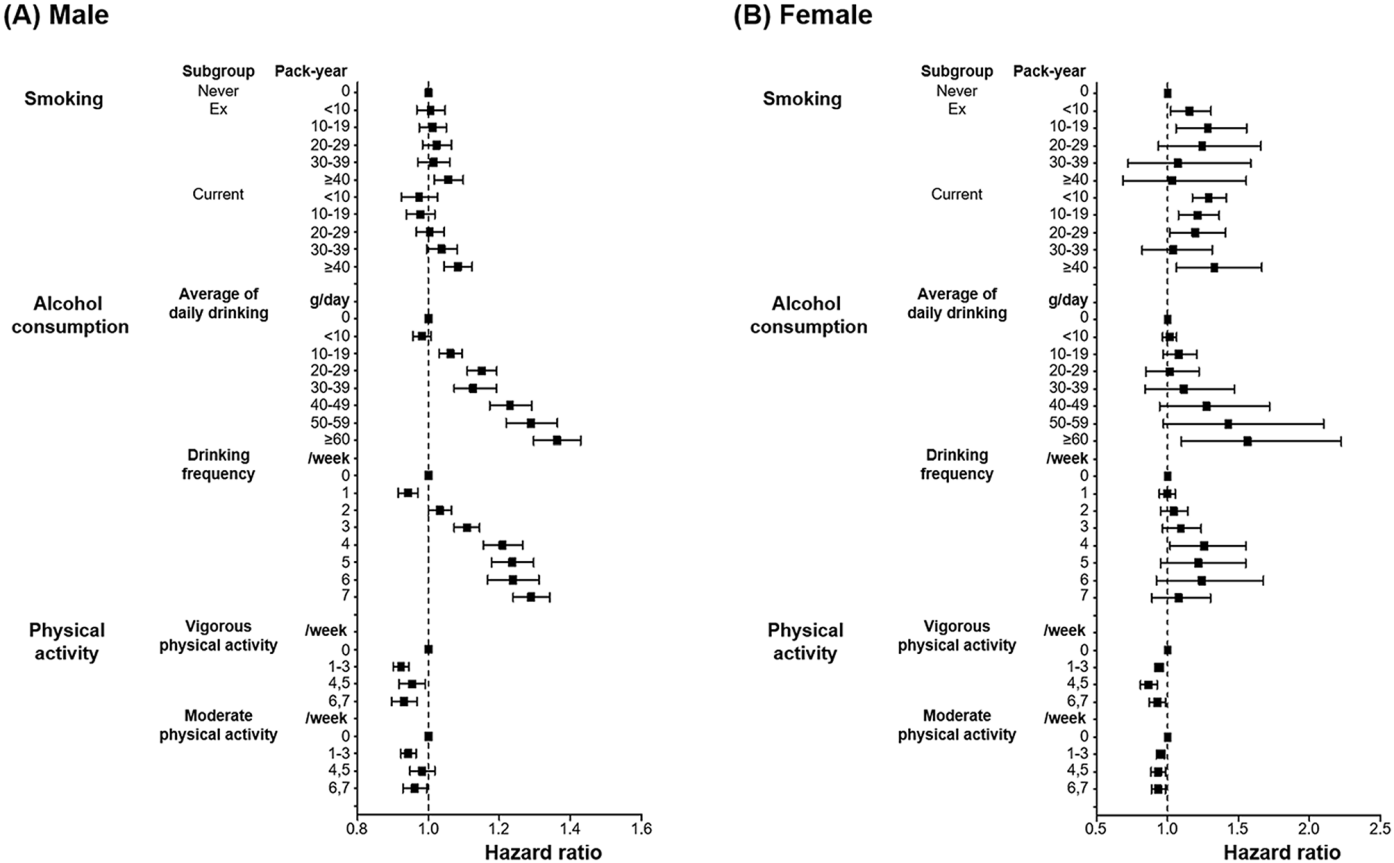
Subgroup analyses of the associations between lifestyle behaviors and new-onset AF. Subgroup analyses according to male (**A**) and female (**B**) are presented. Detailed stratification were additionally performed according to lifestyle behaviors. Smoking was categorized by pack-years, alcohol consumption by average daily alcohol consumption and weekly drinking frequency, and physical activity by the number of moderate- and vigorous-intensity exercise sessions per week. Data regarding lifestyle behaviors were collected during health check-ups as a self-reported questionnaire.

We divided the patients into males and females and investigated the associations between lifestyle behaviors and new-onset AF (Fig. 2 and Supplementary Table 5). Intriguingly, a significant association was observed between sex and smoking (p for interaction < 0.001). Female ex-smokers (HR 1.18, 95% CI 1.08–1.29) and current smokers (HR 1.23, 95% CI 1.16–1.31) demonstrated a substantially higher risk of AF than never smokers. Male ex-smokers (HR 1.03, 95% CI 1.00–1.05), but not current smokers (HR 1.02, 95% CI 1.00–1.05), had an increased risk of AF compared to never smokers. Only males who currently or previously smoked more than 40 pack-years had a higher risk of AF. The subgroup analyses of the cumulative smoking dose are presented in Fig. 3 and Supplementary Table 6.

Conversely, no significant interaction was identified between alcohol consumption and new-onset AF (p for interaction = 0.485). With respect to male subjects, moderate (HR 1.12, 95% CI 1.09–1.15) and heavy drinkers (HR 1.24, 95% CI 1.21–1.28) had a higher risk of AF, and mild drinkers (HR 1.01, 95% CI 0.98–1.03) had an AF risk similar to that of non-drinkers. Regarding female subjects, mild (HR 1.03, 95% CI 0.98–1.08) and moderate drinkers (HR 1.02, 95% CI 0.90–1.15) had a similar AF risk, while heavy drinkers (HR 1.28, 95% CI 1.09–1.51) had a higher AF risk. Figure 3 and Supplementary Table 7 present additional analyses based on more sophisticated stratification methods. In male subjects, a J-shaped association was observed between alcohol consumption and AF risk, but no such association was observed in female subjects.

In both sexes, MVPA was associated with a lower risk of AF (HR 0.93, 95% CI 0.91–0.95 for males; HR 0.93, 95% CI 0.90–0.95 for females). No significant interaction was identified between sex and physical activity (p for interaction = 0.212). The amount of vigorous and moderate exercise showed a linear correlation with AF risk in both sexes (p for trend < 0.01 in both sexes, Fig. 3 and Supplementary Table 8).

### Lifestyle behaviors and AF in patients with DM

**Figure 4:**
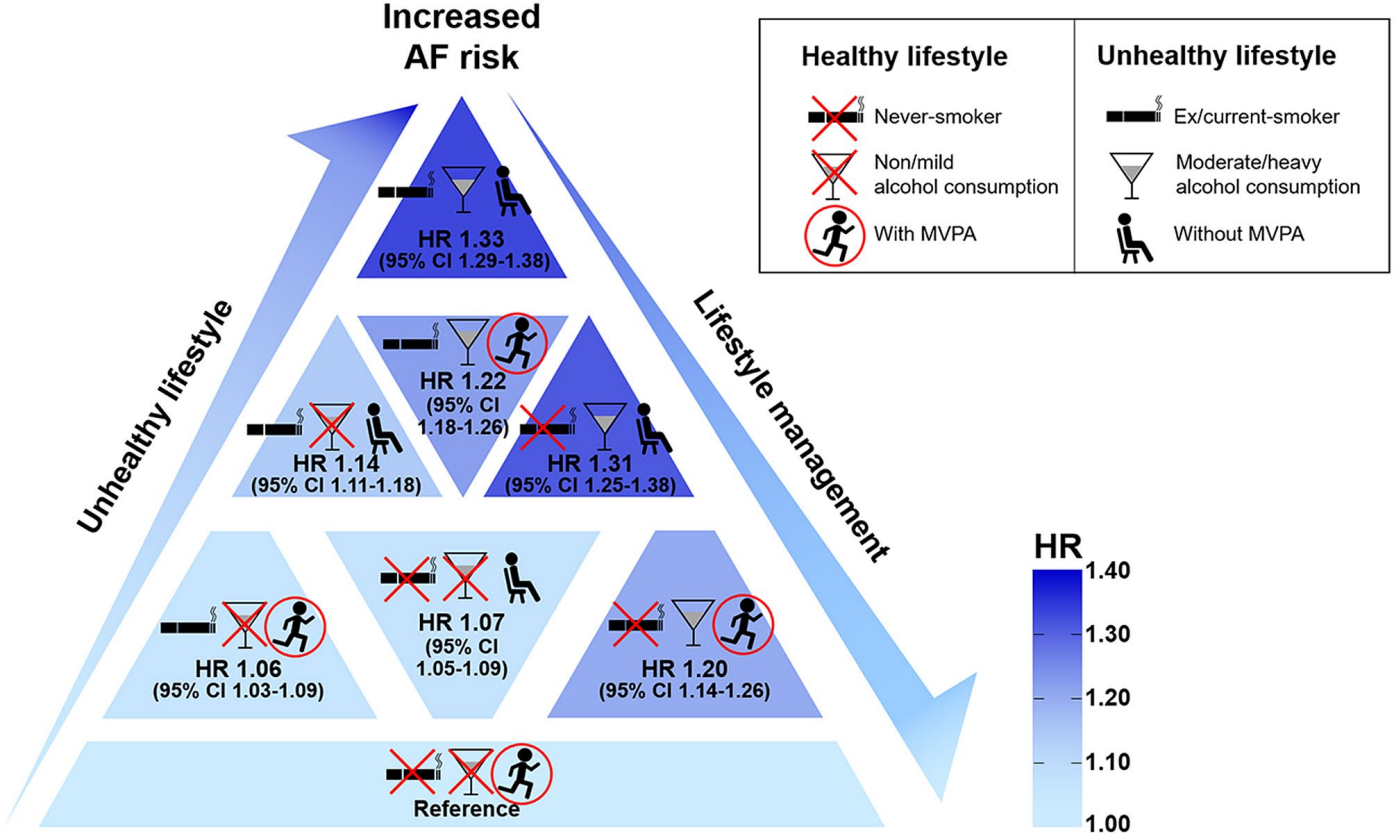
Associations between lifestyle behaviors and AF risk. The percentage of patients and HRs for adjusted AF risk stratified by lifestyle behaviors are presented. Patients who were never smokers, non-/mild drinkers, and engaged in MVPA were considered the reference group. Data regarding lifestyle behaviors were collected during health check-ups as a self-reported questionnaire. *AF* atrial fibrillation, *HR* hazard ratio, *MVPA* moderate-to-vigorous physical activity.

Based on tobacco smoking, alcohol consumption, and physical activity, we classified patients into eight groups and analyzed AF risk (Fig. 4 and Supplementary Table 9). Among the entire study population, 542,271 (21.3%) subjects were never smokers, non-/mild drinkers, and engaged in MVPA. When these patients were regarded as the reference group, all other groups showed a higher risk of AF.

## Discussion

In this study, we comprehensively investigated the associations between lifestyle behaviors and newly developed AF in patients with DM. The main findings of our study were as follows: (1) both ex-smokers and current smokers have a higher risk of AF than never smokers; (2) moderate/heavy drinkers have a higher risk of new-onset AF than non-drinkers, but mild drinkers have a similar risk; (3) patients who engage in MVPA have a lower risk of AF than those who do not; and (4) in the subgroup analyses, a significant interaction was observed between smoking and sex, but not between alcohol consumption or physical activity and sex.

We revealed that DM patients with a history of smoking had a higher risk of AF, which correlates with previous studies that identified smoking as an independent risk factor of AF in other populations^21,22^. Interestingly, there was a significant interaction between sex and smoking for AF incidence (Fig. 2); AF risk was higher in female subjects with a history of smoking than in male subjects with a history of smoking. Indeed, females are known to be more vulnerable to the detrimental cardiovascular effects of smoking such as stroke and myocardial infarction^23,24^. The biological mechanisms underlying this phenomenon remain unknown, but the anti-estrogen and early menopause-inducing effects of smoking may be involved^25^.

Previous studies reported a J-shaped association between alcohol consumption and clinical outcomes^20,26,27^, although whether abstainer bias and the sick quitter phenomenon contribute to this association remains controversial. In this study, mild drinkers showed a similar AF risk, while moderate and heavy drinkers showed a higher AF risk compared to non-drinkers. When subgroup analysis was conducted according to weekly drinking frequency, patients who drank 1 day per week (HR 0.96, 95% CI 0.94–0.99) exhibited a significantly lower risk of AF compared to non-drinkers (Supplementary Table 3), which was consistent with a previous report^26^. Although we did not explore the biological mechanisms underlying this observation, several possible explanations exist. Small quantities of alcohol have been shown to have an anti-inflammatory effect, improve insulin sensitivity, and reduce postprandial glucose excursions in patients with type 2 DM^28,29^, all of which have been reported to be associated with the development of AF.

Given that physical activity has been shown to control blood glucose, manage body weight, and reduce cardiovascular mortality, it is considered an important component of DM management^17,30^. In addition to improving metabolic status, physical activity also maintains arterial elasticity, which is protective against AF^31^. However, as vigorous-intensity exercise can induce left atrial enlargement, inflammation of the atrium, and an increase in parasympathetic tone, the association between physical activity and AF remains controversial^32,33^. In this study, we found that both vigorous and moderate-intensity physical activity are associated with a lower risk of new-onset AF in DM patients.

Type 2 DM is prevalent worldwide, and its social burden has substantially surged^1^. Owing to its pathologic contribution to ASCVD, many attempts have been made to improve clinical outcomes in patients with DM by preventing and managing ASCVD^4^. The incidence of AF, a common and critical arrhythmia, is thought to be increased in patients with DM^13^, but few studies have explored the risk factors of AF in these individuals. In this nationwide study, we demonstrated that lifestyle behaviors, including smoking, alcohol consumption, and physical activity, are significantly associated with the development of AF. Therefore, further efforts to manage these factors in DM patients should be made in the future to manage AF risk in these individuals.

There are several limitations in this study. First, this study was an observational study based on a nationwide claims database. Unmeasured confounders, including the HbA1c level, could not be adjusted. Although we have adjusted age and DM duration in Cox regression analysis, the age difference between AF and non-AF groups were significant, so unmeasured confounders related to age could also influence the results. However, given that randomized trials of smoking and alcohol consumption are unlikely due to ethical issues, carefully managed observational studies could provide clinical implications. Second, our comprehensive investigation mainly dealt with epidemiologic data, and the biological mechanisms underlying the associations between lifestyle behaviors and AF risk were not investigated. Third, because we only analyzed East Asians, the study findings cannot be directly extrapolated to other populations of different ethnicities. Finally, as we did not include type 1 DM patients in this study, the results obtained were not applicable to patients with type 1 DM.

In this large nationwide cohort study, we found that smoking, alcohol consumption, and physical activity were strongly associated with new-onset AF in patients with DM. When subgroup analyses were conducted according to sex, a significant interaction was identified between smoking and sex, but no interaction was observed between alcohol consumption or physical activity and sex. These results suggest that lifestyle management may have valuable therapeutic implications in patients with DM. In addition, in further studies of anti-diabetic therapies, the risk of new-onset AF should be evaluated in addition to that of ASCVD.

## Materials and methods

### Data source and study population

This study used data obtained from the NHIS database. In 2000, the NHIS was established as the single insurer for the entire Korean population. The NHIS also provides regular health check-up programs for the public, and adults are recommended to undergo a check-up at least once every 2 years. Information on the NHIS has been published elsewhere^34^. The NHIS database is available to researchers, and anonymized data are supplied if an official review committee has approved the study protocol. This study was conducted according to the Declaration of Helsinki and was approved by the Institutional Review Board of Seoul National University Hospital (IRB No. E-1908-027-1052).

There are various data subsets of the NHIS database, including the qualification, claim, health check-up, and death information databases^35^. Together, these databases comprise comprehensive information about demographics, disease diagnosis (according to the 10th revision of the International Classification of Diseases [ICD-10] codes), laboratory exams and imaging studies, medical treatments, and hospitalization.

We screened 2,671,490 DM patients older than 30 years between 2009 and 2012. From this initial population, we excluded 63,015 subjects whose data on lifestyle behaviors was not available. In addition, we excluded further 57,439 individuals who had a previous history of AF. Therefore, 2,551,036 subjects were ultimately included and followed up to 2017.

### Definitions of diabetes mellitus, lifestyle, and other covariates

Patients with type 2 DM were defined as follows: (1) patients with claims for the ICD-10 code for type 2 DM (I11-I14) and claims for anti-diabetic drug prescriptions (sulfonylureas, metformin, meglitinides, thiazolidinediones, dipeptidyl peptidase-4 inhibitors, α-glucosidase inhibitors, or insulin) or (2) patients who underwent a fasting glucose level test (≥ 126 mg/dL) during a health check-up and were newly diagnosed with type 2 DM (ICD-10 code I11-I14)^36^.

Self-reported data on smoking, alcohol consumption and physical activity were collected during health check-ups. With respect to tobacco smoking, patients were stratified into never-smokers (smoked fewer than 100 cigarettes during their life), ex-smokers, and current smokers^37^. Cumulative smoking was quantified in pack-years. With respect to alcohol consumption, average alcohol intake per day (g/day) and drinking frequency per week were analyzed. The patients were categorized into non-, mild (< 15 g/day), moderate (15–29 g/day), and heavy (≥ 30 g/day) drinkers^38^. With respect to physical activity, the number of sessions of moderate- and vigorous-intensity physical activity per week (/week) undertaken by each patient was surveyed^39^. Moderate-intensity exercise for at least 30 min/day and vigorous-intensity exercise for at least 20 min/day were combined as moderate-to-vigorous physical activity (MVPA).

The definitions of other covariates described in our previous reports are summarized in Supplementary Table 10^40,41^.

### The study endpoint

The study endpoint was newly diagnosed atrial fibrillation (AF, ICD-10 code I48.0–48.4, I48.9) during the admission or more than two times in the outpatient clinic^36^. After the index day of health check-up, subjects were followed for incident AF or until the end of the study period (December 2017), whichever came first.

### Statistical analysis

Data are presented as numbers and frequencies for categorical variables and mean ± standard deviation for continuous variables. For comparisons between groups, the chi-square test (or Fisher’s exact test when expected cell counts were < 5 for a 2 × 2 table) was used for categorical variables, and an unpaired Student’s t-test was used for continuous variables. The annual event incidence rates (IR) were calculated as the number of events per 1000 person-years (PY). Multivariate Cox proportional hazard regression models were used to estimate hazard ratios (HRs) and corresponding 95% confidence intervals (CIs) for the associations between lifestyle behaviors and newly developed AF. The models used adjusted for a number of covariates: model 1 adjusted for age and sex; model 2 additionally adjusted for income level, body mass index, a previous history of hypertension and hyperlipidemia, DM duration, and a history of past anti-diabetic medication; and model 3 additionally adjusted for the other lifestyle behaviors (smoking, alcohol consumption, and MVPA). Subgroup analyses were conducted according to sex using Cox models. Two-sided *P* values < 0.05 were considered statistically significant. All statistical analyses were performed using SAS version 9.4 (SAS Institute, Cary, North Carolina, USA).

## Supplementary Information

Supplementary Information.
