# Supplementary material for: Lifestyle is associated with atrial fibrillation development in patients with type 2 diabetes mellitus
Source: Sci Rep. 2021 Feb 25;11:4676. doi: 10.1038/s41598-021-84307-5 (PMC7907194; doi:10.1038/s41598-021-84307-5)
Supplement: Supplementary file 1 — Supplementary Information. [file 41598_2021_84307_MOESM1_ESM.docx]

**Lifestyle is Associated with Atrial Fibrillation Development in Patients with Type 2 Diabetes Mellitus**

Chan Soon Park,^1,2,*^ Kyung-Do Han,^3,*^ Eue-Keun Choi,^1*^ Da Hye Kim,^3^ Hyun-Jung Lee,^1^ So-Ryoung Lee,^1^ Seil Oh^1^

^1^Department of Internal Medicine, Seoul National University Hospital, Seoul, Republic of Korea

^2^Graduate School of Medical Science and Engineering, Korea Advanced Institute of Science and Technology, Daejeon, Republic of Korea

^3^Department of Biostatistics, College of Medicine, The Catholic University of Korea, Seoul, Korea

* Park CS and Han K contributed equally to this work.

**Short tile**: Lifestyle and AF in DM patients

**Corresponding Author:**

Eue-Keun Choi, MD, PhD

Professor, Department of Internal Medicine, Seoul National University Hospital,

101 Daehak-ro, Jongno-gu, Seoul, 03080, Republic of Korea

Tel: 82-2-2072-0688

Fax: 82-2-762-9662

E-mail: [choiek17@snu.ac.kr](mailto:choiek17@snu.ac.kr)

**Supplementary material**

**
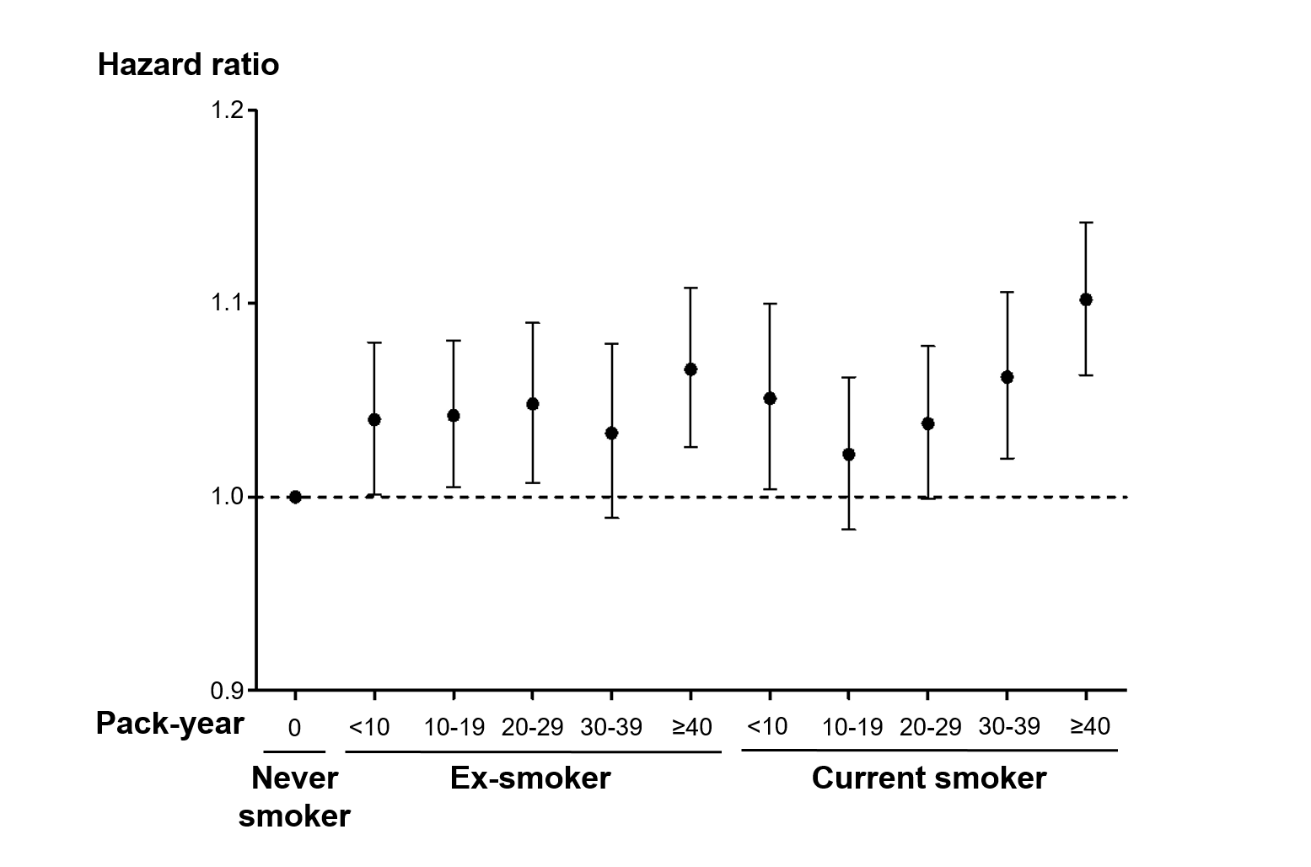
**

**Supplementary figure 1.** Association between smoking history and risk of new-onset atrial fibrillation in patients with DM

Risk of atrial fibrillation according to smoking status and accumulative dose was presented. Data regarding smoking history were collected during health check-ups as a self-reported questionnaire.

**
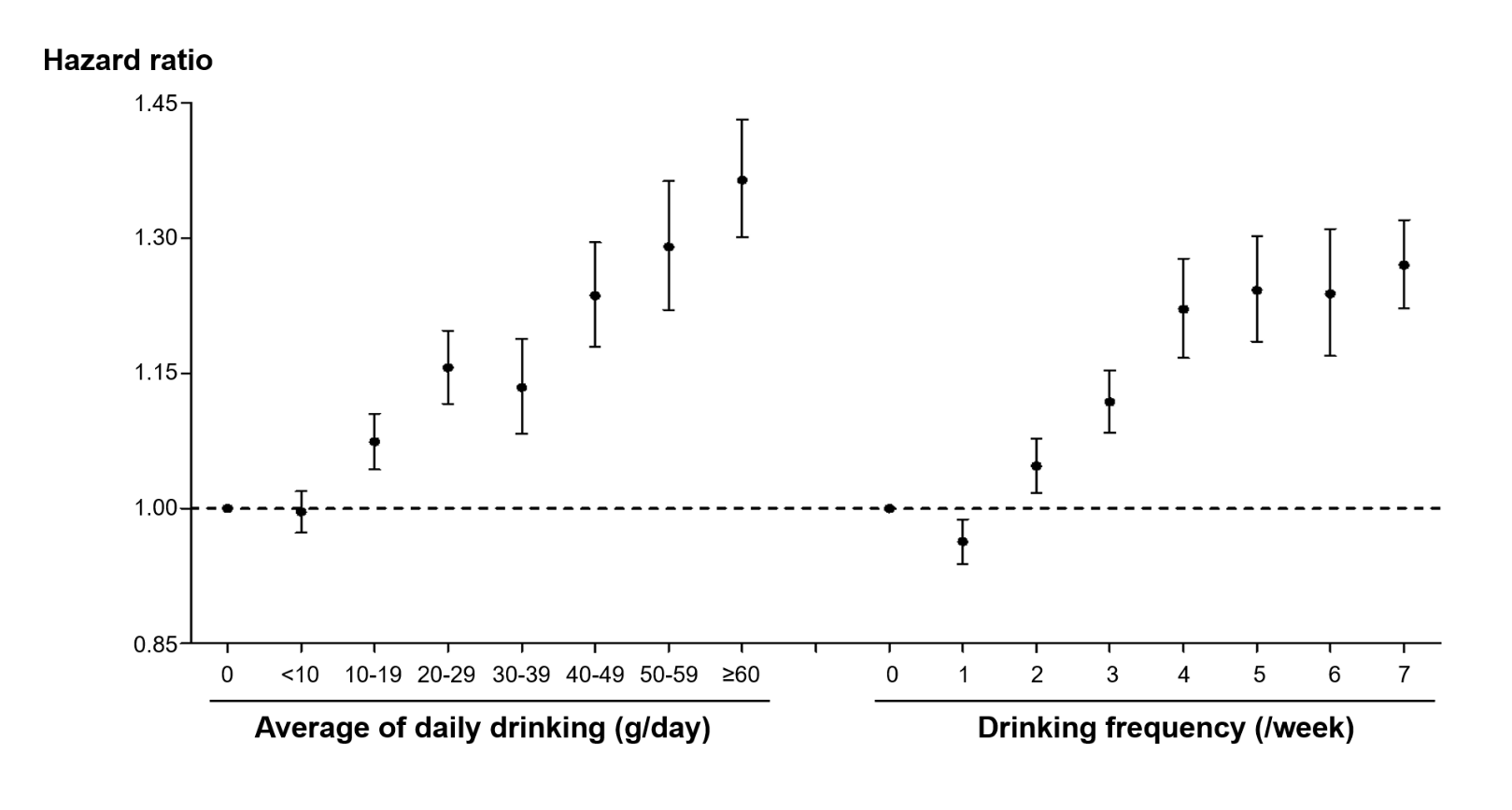
**

**Supplementary figure 2.** Association between drinking habits and risk of new-onset atrial fibrillation in patients with DM

Risk of atrial fibrillation according to the average of daily drinking and drinking frequency was presented. Data regarding alcohol consumption were collected during health check-ups as a self-reported questionnaire.

**
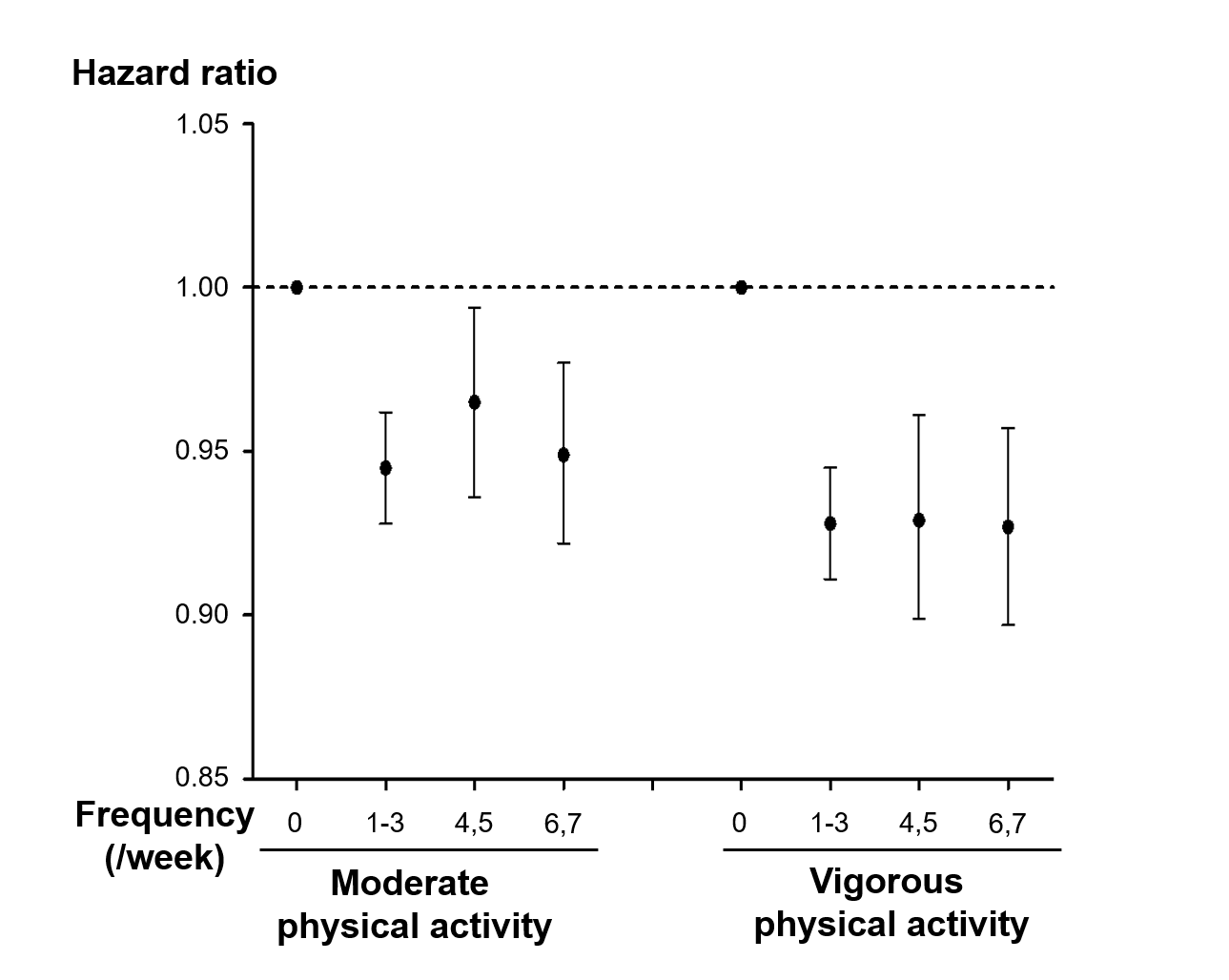
**

**Supplementary figure 3.** Association between physical activity and risk of new-onset atrial fibrillation in patients with DM

Risk of atrial fibrillation according to the intensity and frequency of physical activity was presented. Data regarding physical activity were collected during health check-ups as a self-reported questionnaire.

**Supplementary Table 1**. Risk of AF according to lifestyle in DM patients

| **Lifestyle** | **Number of patients** | **AF events** | **Follow-up duration** | **IR*** | **HR (95% CI)**** | | |
| --- | --- | --- | --- | --- | --- | --- | --- |
|  |  |  |  |  | **Model 1** | **Model 2** | **Model 3** |
| **Smoking** | | | | | | | |
| Never | 1,427,015 | 44,058 | 10,176,769.0 | 4.33 | 1 (reference) | 1 (reference) | 1 (reference) |
| Ex | 467,444 | 14,914 | 3,289,775.2 | 4.53 | 1.06 (1.04-1.08) | 1.06 (1.03-1.08) | 1.05 (1.02-1.07) |
| Current | 656,577 | 15,016 | 4,608,973.0 | 3.26 | 1.05 (1.03-1.07) | 1.09 (1.07-1.12) | 1.06 (1.03-1.08) |
| P for trend |  |  |  |  | <0.001 | <0.001 | <0.001 |
| **Alcohol consumption** | | | | | | | |
| Non | 1,462,406 | 46,887 | 10,409,857.0 | 4.50 | 1 (reference) | 1 (reference) | 1 (reference) |
| Mild | 546,618 | 13,009 | 3,852,357.2 | 3.38 | 1.00 (0.98-1.02) | 1.01 (0.99-1.03) | 1.01 (0.99-1.03) |
| Moderate | 286,117 | 6,945 | 2,013,661.8 | 3.45 | 1.12 (1.09-1.15) | 1.12 (1.09-1.16) | 1.12 (1.09-1.15) |
| Heavy | 255,895 | 7,147 | 1,799,641.3 | 3.97 | 1.26 (1.23-1.29) | 1.26 (1.22-1.29) | 1.24 (1.21-1.28) |
| P for trend |  |  |  |  | <0.001 | <0.001 | <0.001 |
| **Physical activity** | | | | | | | |
| no MVPA | 1,332,830 | 43,433 | 9,423,631.7 | 4.61 | 1 (reference) | 1 (reference) | 1 (reference) |
| MVPA | 1,218,206 | 30,555 | 8,651,885.5 | 3.53 | 0.92 (0.91-0.94) | 0.93 (0.91-0.94) | 0.93 (0.91-0.94) |
| P for trend |  |  |  |  | <0.001 | <0.001 | <0.001 |

*Incidence rates were calculated per 1,000 patient-years from baseline population

**Model 1 adjusts age and sex; Model 2 adjusts Model 1 with income level, body mass index, previous history of hypertension and hyperlipidemia, duration and previous medication for diabetes mellitus; Model 3 adjusts Model 2 with smoking, alcohol consumption and MVPA

AF, atrial fibrillation; CI, confidence interval; DM, diabetes mellitus; IR, incidence rate; MVPA, moderate-to-vigorous physical activity; PY, pack-year

**Supplementary Table 2**. Risk of AF stratified by smoking status and accumulative dose in patients with DM

| **Smoking status** | **Amount**  **(PY)** | **Number of patients** | **AF events** | **Follow-up duration** | **IR*** | **HR (95% CI)**** | | |
| --- | --- | --- | --- | --- | --- | --- | --- | --- |
|  |  |  |  |  |  | **Model 1** | **Model 2** | **Model 3** |
| Non | 0 | 1,427,015 | 44,058 | 10,176,769.0 | 4.33 | 1(ref.) | 1(ref.) | 1(ref.) |
| Ex | <10 | 122,008 | 3,204 | 854,604.2 | 3.75 | 1.03 (0.99-1.07) | 1.04 (1.00-1.08) | 1.04 (1.00-1.08) |
|  | 10-19 | 121,302 | 3,437 | 853,094.4 | 4.03 | 1.04 (1.01-1.08) | 1.05 (1.01-1.09) | 1.04 (1.01-1.08) |
|  | 20-29 | 92,241 | 2,888 | 649,868.9 | 4.44 | 1.06 (1.02-1.11) | 1.06 (1.02-1.10) | 1.05 (1.01-1.09) |
|  | 30-39 | 63,399 | 2,292 | 447,789.8 | 5.12 | 1.06 (1.01-1.11) | 1.05 (1.01-1.10) | 1.03 (0.99-1.08) |
|  | ≥40 | 68,494 | 3,093 | 484,417.9 | 6.39 | 1.11 (1.07-1.15) | 1.09 (1.05-1.14) | 1.07 (1.03-1.11) |
| Current | <10 | 118,264 | 2,063 | 821,341.0 | 2.51 | 1.02 (0.98-1.07) | 1.06 (1.02-1.11) | 1.05 (1.00-1.10) |
|  | 10-19 | 170,929 | 3,110 | 1,195,890.5 | 2.60 | 0.99 (0.96-1.03) | 1.04 (1.00-1.08) | 1.02 (0.98-1.06) |
|  | 20-29 | 152,318 | 3,259 | 1,070,601.3 | 3.04 | 1.02 (0.99-1.06) | 1.07 (1.03-1.11) | 1.04 (1.00-1.08) |
|  | 30-39 | 113,325 | 2,830 | 798,473.1 | 3.54 | 1.07 (1.03-1.11) | 1.11 (1.07-1.16) | 1.06 (1.02-1.11) |
|  | ≥40 | 101,741 | 3,754 | 722,667.1 | 5.20 | 1.13 (1.09-1.17) | 1.16 (1.12-1.21) | 1.10 (1.06-1.14) |
| P for trend |  |  |  |  |  | <0.001 | <0.001 | <0.001 |

*Incidence rates were calculated per 1,000 patient-years from baseline population

**Model 1 adjusts age and sex; Model 2 adjusts Model 1 + income level, body mass index, previous history of hypertension and hyperlipidemia, duration and previous medication for diabetes mellitus; Model 3 adjusts Model 2 + alcohol consumption and moderate-to-severe physical activity

AF, atrial fibrillation; CI, confidence interval; DM, diabetes mellitus; HR, hazard ratio; IR, incidence rate; PY, pack-year

**Supplementary Table 3**. Risk of AF stratified by drinking habits in patients with DM

| **Drinking habit** | | **Number of patients** | **AF events** | **Follow-up duration** | **IR*** | **HR (95% CI)**** | | |
| --- | --- | --- | --- | --- | --- | --- | --- | --- |
|  |  |  |  |  |  | **Model 1** | **Model 2** | **Model 3** |
| **Average of daily drinking (g/day)** | | | |  |  |  |  |  |
| 0 | | 1,462,406 | 46,887 | 10,409,857.0 | 4.50 | 1 (reference) | 1 (reference) | 1 (reference) |
| <10 | | 420,091 | 9,966 | 2,962,860.8 | 3.36 | 0.98 (0.96-1.00) | 0.99 (0.97-1.02) | 1.00 (0.97-1.02) |
| 10-19 | | 255,593 | 6,106 | 1,800,181.2 | 3.39 | 1.07 (1.04-1.10) | 1.08 (1.05-1.11) | 1.07 (1.04-1.11) |
| 20-29 | | 157,051 | 3,882 | 1,102,977.0 | 3.52 | 1.16 (1.12-1.20) | 1.16 (1.12-1.20) | 1.16 (1.12-1.20) |
| 30-39 | | 87,671 | 1,998 | 616,885.1 | 3.24 | 1.15 (1.09-1.20) | 1.14 (1.09-1.20) | 1.13 (1.08-1.19) |
| 40-49 | | 68,020 | 1,926 | 477,033.8 | 4.04 | 1.25 (1.19-1.31) | 1.25 (1.19-1.31) | 1.24 (1.18-1.30) |
| 50-59 | | 40,171 | 1,345 | 282,548.9 | 4.76 | 1.30 (1.23-1.37) | 1.31 (1.24-1.38) | 1.29 (1.22-1.36) |
| ≥60 | | 60,033 | 1,878 | 423,173.5 | 4.44 | 1.39 (1.33-1.46) | 1.38 (1.32-1.45) | 1.36 (1.30-1.43) |
| P for trend | |  |  |  |  | <0.001 | <0.001 | <0.001 |
| **Drinking frequency (/week)** | | | |  |  |  |  |  |
| 0 | | 1,462,406 | 46,887 | 10,409,857.0 | 4.50 | 1 (reference) | 1 (reference) | 1 (reference) |
| 1 | | 361,979 | 7,420 | 2,558,652.3 | 2.89 | 0.95 (0.93-0.97) | 0.96 (0.94-0.99) | 0.96 (0.94-0.99) |
| 2 | | 282,448 | 6,260 | 1,989,403.4 | 3.15 | 1.04 (1.01-1.07) | 1.05 (1.02-1.08) | 1.05 (1.02-1.08) |
| 3 | | 206,599 | 5,151 | 1,449,290.3 | 3.55 | 1.12 (1.09-1.16) | 1.12 (1.09-1.16) | 1.12 (1.08-1.15) |
| 4 | | 75,815 | 2,142 | 531,335.1 | 4.03 | 1.22 (1.17-1.28) | 1.23 (1.18-1.29) | 1.22 (1.17-1.28) |
| 5 | | 61,362 | 1,909 | 428,797.3 | 4.45 | 1.25 (1.19-1.31) | 1.25 (1.19-1.31) | 1.24 (1.19-1.30) |
| 6 | | 34,383 | 1,246 | 242,058.6 | 5.15 | 1.23 (1.16-1.30) | 1.25 (1.18-1.32) | 1.24 (1.17-1.31) |
| 7 | | 66,044 | 2,973 | 466,123.3 | 6.38 | 1.26 (1.21-1.31) | 1.29 (1.24-1.34) | 1.27 (1.22-1.32) |
| P for trend |  |  |  |  |  | <0.001 | <0.001 | <0.001 |

*Incidence rates were calculated per 1,000 patient-years from baseline population

**Model 1 adjusts age and sex; Model 2 adjusts Model 1 + income level, body mass index, previous history of hypertension and hyperlipidemia, duration and previous medication for diabetes mellitus; Model 3 adjusts Model 2 + smoking and moderate-to-severe physical activity

AF, atrial fibrillation; CI, confidence interval; DM, diabetes mellitus; HR, hazard ratio; IR, incidence rate

**Supplementary Table 4**. Risk of AF stratified by physical activity intensity and frequency in patients with DM

| **Physical activity** | **Frequency**  **(days/week)** | **Number of patients** | **AF events** | **Follow-up duration** | **IR*** | **HR (95% CI)**** | | |
| --- | --- | --- | --- | --- | --- | --- | --- | --- |
|  |  |  |  |  |  | **Model 1** | **Model 2** | **Model 3** |
| Moderate | 0 | 1,618,378 | 51,254 | 11,443,297.4 | 4.48 | 1 (reference) | 1 (reference) | 1 (reference) |
|  | 1-3 | 667,033 | 14,986 | 4,727,191.4 | 3.17 | 0.92 (0.90-0.94) | 0.93 (0.91-0.95) | 0.93 (0.91-0.95) |
|  | 4,5 | 143,859 | 3,725 | 1,027,098.6 | 3.63 | 0.93 (0.90-0.96) | 0.93 (0.90-0.96) | 0.93 (0.90-0.96) |
|  | 6,7 | 121,766 | 4,023 | 877,929.8 | 4.58 | 0.93 (0.90-0.96) | 0.93 (0.90-0.96) | 0.93 (0.90-0.96) |
| P for trend |  |  |  |  |  | <0.001 | <0.001 | <0.001 |
| Vigorous | 0 | 1,506,048 | 47,549 | 10,661,814.8 | 4.46 | 1 (reference) | 1 (reference) | 1 (reference) |
|  | 1-3 | 711,595 | 16,501 | 5,034,707.1 | 3.28 | 0.94 (0.92-0.96) | 0.94 (0.93-0.96) | 0.95 (0.93-0.96) |
|  | 4,5 | 179,602 | 4,839 | 1,277,336.2 | 3.79 | 0.96 (0.93-0.99) | 0.96 (0.94-0.99) | 0.97 (0.94-0.99) |
|  | 6,7 | 153,791 | 5,099 | 1,101,659.2 | 4.63 | 0.95 (0.92-0.98) | 0.95 (0.92-0.98) | 0.95 (0.92-0.98) |
| P for trend |  |  |  |  |  | <0.001 | <0.001 | <0.001 |

*Incidence rates were calculated per 1,000 patient-years from baseline population

**Model 1 adjusts age and sex; Model 2 adjusts Model 1 + income level, body mass index, previous history of hypertension and hyperlipidemia, duration and previous medication for diabetes mellitus; Model 3 adjusts Model 2 + smoking and alcohol consumption

AF, atrial fibrillation; CI, confidence interval; DM, diabetes mellitus; HR, hazard ratio; IR, incidence rate, MVPA, moderate-to-vigorous physical activity

**Supplementary Table 5**. Sex differences of association between smoking status, accumulative dose, and AF in patients with DM

| **Lifestyle** | **Male** | | | |  | **Female** | | | |
| --- | --- | --- | --- | --- | --- | --- | --- | --- | --- |
|  | **Number of patients** | **AF Events** | **Follow-up duration** | **HR (95% CI)*** |  | **Number of patients** | **AF Events** | **Follow-up duration** | **HR (95% CI)*** |
| **Smoking** | | | | | | | | | |
| Never | 455,152 | 15,910 | 3,244,686.7 | 1 (reference) |  | 971,863 | 28,148 | 6,932,082.3 | 1 (reference) |
| Ex | 453,094 | 14,456 | 3,190,901.4 | 1.03 (1.00-1.05) |  | 14,350 | 458 | 98,873.8 | 1.18 (1.08-1.29) |
| Current | 618,895 | 13,986 | 4,348,133.7 | 1.02 (1.00-1.05) |  | 37,682 | 1,030 | 260,839.3 | 1.23 (1.16-1.31) |
| P for trend |  |  |  | 0.076 |  |  |  |  | <0.001 |
| **Alcohol consumption** | | | | | | | | | |
| Non | 567,447 | 19,587 | 4,020,193.8 | 1 (reference) |  | 894,959 | 27,300 | 6,389,663.2 | 1 (reference) |
| Mild | 441,403 | 11,079 | 3,114,385.6 | 1.01 (0.98-1.03) |  | 105,215 | 1,930 | 737,971.6 | 1.03 (0.98-1.08) |
| Moderate | 269,945 | 6,689 | 1,901,536.5 | 1.12 (1.09-1.15) |  | 16,172 | 256 | 112,125.3 | 1.02 (0.90-1.15) |
| Heavy | 248,346 | 6,997 | 1,747,606.0 | 1.24 (1.21-1.28) |  | 7,549 | 150 | 52,035.4 | 1.28 (1.09-1.51) |
| P for trend |  |  |  | <0.001 |  |  |  |  | 0.014 |
| **Physical activity** | | | | | | | | | |
| no MVPA | 1,175,365 | 33,573 | 8,275,895.2 | 1 (reference) |  | 847,736 | 25,499 | 6,026,915.6 | 1 (reference) |
| MVPA | 351,776 | 10,779 | 2,507,826.6 | 0.93 (0.91-0.95) |  | 176,159 | 4,137 | 1,264,879.8 | 0.93 (0.90-0.95) |
| P for trend |  |  |  | <0.001 |  |  |  |  | <0.001 |

*HR is adjusted for age, sex, income level, body mass index, previous history of hypertension and hyperlipidemia, duration and previous medication for diabetes mellitus, smoking, alcohol consumption, and physical activity

AF, atrial fibrillation; CI, confidence interval; DM, diabetes mellitus; HR, hazard ratio; PY, pack-year

**Supplementary Table 6**. Sex differences of association between smoking status, accumulative dose, and AF in patients with DM

| **Smoking status** | **Amount**  **(PY)** | **Male** | | | |  | **Female** | | | |
| --- | --- | --- | --- | --- | --- | --- | --- | --- | --- | --- |
|  |  | **Number of patients** | **AF Events** | **Follow-up duration** | **HR (95% CI)*** |  | **Number of patients** | **AF Events** | **Follow-up duration** | **HR (95% CI)*** |
| Never | 0 | 455,152 | 15,910 | 3244686.7 | 1 (reference) |  | 971,863 | 28,148 | 6932082.3 | 1 (reference) |
| Ex | <10 | 112,378 | 2,945 | 788466.3 | 1.01 (0.97-1.05) |  | 9,630 | 259 | 66137.9 | 1.16 (1.02-1.31) |
|  | 10-19 | 118,770 | 3,333 | 835589.2 | 1.02 (0.98-1.06) |  | 2,532 | 104 | 17505.2 | 1.28 (1.06-1.56) |
|  | 20-29 | 91,143 | 2,841 | 642275.3 | 1.03 (0.99-1.07) |  | 1,098 | 47 | 7593.7 | 1.24 (0.93-1.66) |
|  | 30-39 | 62,805 | 2,267 | 443602.9 | 1.02 (0.97-1.06) |  | 594 | 25 | 4187.0 | 1.07 (0.72-1.59) |
|  | ≥40 | 67,998 | 3,070 | 480967.8 | 1.06 (1.02-1.10) |  | 496 | 23 | 3450.2 | 1.03 (0.68-1.55) |
| Current | <10 | 98,825 | 1,604 | 687638.6 | 0.98 (0.93-1.03) |  | 19,439 | 459 | 133702.4 | 1.29 (1.17-1.41) |
|  | 10-19 | 161,117 | 2,829 | 1127653.1 | 0.98 (0.94-1.02) |  | 9,812 | 281 | 68237.4 | 1.21 (1.08-1.36) |
|  | 20-29 | 147,850 | 3,115 | 1039452.0 | 1.00 (0.97-1.04) |  | 4,468 | 144 | 31149.3 | 1.19 (1.01-1.40) |
|  | 30-39 | 110,980 | 2,761 | 782117.1 | 1.03 (0.99-1.08) |  | 2,345 | 69 | 16356.1 | 1.04 (0.82-1.31) |
|  | ≥40 | 100,123 | 3,677 | 711272.8 | 1.08 (1.04-1.12) |  | 1,618 | 77 | 11394.2 | 1.32 (1.06-1.66) |
| P for trend |  |  |  |  | 0.017 |  |  |  |  | <0.001 |

*HR is adjusted for age, sex, income level, body mass index, previous history of hypertension and hyperlipidemia, duration and previous medication for diabetes mellitus, alcohol consumption, and physical activity

AF, atrial fibrillation; CI, confidence interval; DM, diabetes mellitus; HR, hazard ratio; PY, pack-year

**Supplementary Table 7**. Sex differences of association between drinking habits and AF in patients with DM

| **Drinking habit** | **Male** | | | |  | **Female** | | | |  |
| --- | --- | --- | --- | --- | --- | --- | --- | --- | --- | --- |
|  | **Number of patients** | **AF Events** | **Follow-up duration** | **HR (95% CI)*** |  | **Number of patients** | **AF Events** | **Follow-up duration** | **HR (95% CI)*** | |
| **Average of daily drinking (g/day)** | | |  |  |  |  |  |  |  | |
| 0 | 1,462,406 | 46,887 | 10,409,857.0 | 1 (reference) |  | 894,959 | 27,300 | 6,389,663.2 | 1 (reference) | |
| <10 | 420,091 | 9,966 | 2,962,860.8 | 0.99 (0.96-1.02) |  | 94,110 | 1,735 | 661,024.0 | 1.02 (0.97-1.07) | |
| 10-19 | 255,593 | 6,106 | 1,800,181.2 | 1.07 (1.04-1.10) |  | 19,752 | 334 | 137,056.2 | 1.08 (0.97-1.21) | |
| 20-29 | 157,051 | 3,882 | 1,102,977.0 | 1.16 (1.12-1.20) |  | 7,525 | 117 | 52,016.7 | 1.02 (0.85-1.22) | |
| 30-39 | 87,671 | 1,998 | 616,885.1 | 1.13 (1.08-1.19) |  | 3,008 | 50 | 20,754.6 | 1.11 (0.84-1.47) | |
| 40-49 | 68,020 | 1,926 | 477,033.8 | 1.23 (1.18-1.29) |  | 2,157 | 43 | 14,862.7 | 1.27 (0.94-1.71) | |
| 50-59 | 40,171 | 1,345 | 282,548.9 | 1.29 (1.22-1.36) |  | 1,045 | 26 | 7,224.4 | 1.43 (0.97-2.10) | |
| ≥60 | 60,033 | 1,878 | 423,173.5 | 1.36 (1.30-1.43) |  | 1,339 | 31 | 9,193.7 | 1.56 (1.10-2.22) | |
| P for trend |  |  |  | <0.001 |  |  |  |  | 0.002 | |
| **Drinking frequency (/week)** | | |  |  |  |  |  |  |  | |
| 0 | 567,447 | 19,587 | 4,020,193.8 | 1 (reference) |  | 894,959 | 27,300 | 6,389,663.2 | 1 (reference) | |
| 1 | 287,664 | 6,114 | 2,035,733.0 | 0.95 (0.92-0.978) |  | 74,315 | 1,306 | 522,919.3 | 1.00 (0.95-1.06) | |
| 2 | 254,647 | 5,786 | 1,796,055.9 | 1.04 (1.01-1.072) |  | 27,801 | 474 | 193,347.6 | 1.05 (0.96-1.15) | |
| 3 | 192,356 | 4,896 | 1,350,944.0 | 1.11 (1.08-1.151) |  | 14,243 | 255 | 98,346.3 | 1.09 (0.97-1.24) | |
| 4 | 71,843 | 2,055 | 503,912.9 | 1.21 (1.16-1.271) |  | 3,972 | 87 | 27,422.2 | 1.26 (1.02-1.55) | |
| 5 | 58,364 | 1,844 | 408,065.5 | 1.24 (1.18-1.301) |  | 2,998 | 65 | 20,731.8 | 1.22 (0.95-1.55) | |
| 6 | 32,802 | 1,203 | 231,031.1 | 1.24 (1.17-1.314) |  | 1,581 | 43 | 11,027.5 | 1.24 (0.92-1.67) | |
| 7 | 62,018 | 2,867 | 437,785.7 | 1.28 (1.24-1.34) |  | 4,026 | 106 | 28,337.6 | 1.07 (0.89-1.30) | |
| P for trend |  |  |  | <0.001 |  |  |  |  | 0.005 | |

*HR is adjusted for age, sex, income level, body mass index, previous history of hypertension and hyperlipidemia, duration and previous medication for diabetes mellitus, alcohol consumption, and physical activity

AF, atrial fibrillation; CI, confidence interval; DM, diabetes mellitus; HR, hazard ratio; PY, pack-year

**Supplementary Table 8**. Sex differences of association between physical activity intensity, frequency, and AF in patients with DM

| **Physical activity** | **Frequency**  **(days/week)** | **Male** | | | |  | **Female** | | | |
| --- | --- | --- | --- | --- | --- | --- | --- | --- | --- | --- |
|  |  | **Number of patients** | **AF Events** | **Follow-up duration** | **HR (95% CI)*** |  | **Number of patients** | **AF Events** | **Follow-up duration** | **HR (95% CI)*** |
| Moderate | 0 | 823,574 | 26,042 | 5,804,755.5 | 1 (reference) |  | 682,474 | 21,507 | 4,857,059.3 | 1 (reference) |
|  | 1-3 | 489,705 | 11,273 | 3,458,243.0 | 0.94 (0.92-0.97) |  | 221,890 | 5,228 | 1,576,464.1 | 0.95 (0.921-0.98) |
|  | 4,5 | 116,958 | 3,442 | 830,029.0 | 0.98 (0.95-1.02) |  | 62,644 | 1,397 | 447,307.2 | 0.93 (0.884-0.99) |
|  | 6,7 | 96,904 | 3,595 | 690,694.3 | 0.96 (0.93-1.00) |  | 56,887 | 1,504 | 410,964.9 | 0.93 (0.886-0.98) |
| P for trend |  |  |  |  | 0.001 |  |  |  |  | <0.001 |
| Vigorous | 0 | 858,716 | 27,630 | 6,040,675.5 | 1 (reference) |  | 759,662 | 23,624 | 5,402,621.9 | 1 (reference) |
|  | 1-3 | 486,558 | 10,907 | 3,442,066.5 | 0.92 (0.90-0.94) |  | 180,475 | 4,079 | 1,285,124.9 | 0.94 (0.91-0.97) |
|  | 4,5 | 99,190 | 2,823 | 707,362.0 | 0.95 (0.92-0.99) |  | 44,669 | 902 | 319,736.6 | 0.87 (0.81-0.93) |
|  | 6,7 | 82,677 | 2,992 | 593,617.8 | 0.93 (0.90-0.97) |  | 39,089 | 1,031 | 284,312.1 | 0.93 (0.87-0.99) |
| P for trend |  |  |  |  | <0.001 |  |  |  |  | <0.001 |

*HR is adjusted for age, sex, income level, body mass index, previous history of hypertension and hyperlipidemia, duration and previous medication for diabetes mellitus, smoking, and alcohol consumption

AF, atrial fibrillation; CI, confidence interval; DM, diabetes mellitus; HR, hazard ratio

**Supplementary Table 9. Risk of AF stratified by lifestyle behaviors in patients with DM**

| **Smoking status** | **Drinking habit** | **Physical activity** | **Number of patients** | **AF Events** | **Follow-up duration** | **IR*** | **HR (95% CI)**** |
| --- | --- | --- | --- | --- | --- | --- | --- |
| Never | Non/mild | MVPA | 542,271 | 14,204 | 3,877,902.6 | 3.66 | 1 (reference) |
|  |  | no MVPA | 776,938 | 26,407 | 5,533,351.6 | 4.77 | 1.07 (1.05-1.09) |
|  | Moderate/heavy | MVPA | 56,820 | 1,633 | 404,502.5 | 4.04 | 1.20 (1.14-1.26) |
|  |  | no MVPA | 50,986 | 1,814 | 361,012.4 | 5.02 | 1.31 (1.25-1.38) |
| Ex/Current | Non/mild | MVPA | 377,643 | 9,283 | 2,667,040.7 | 3.48 | 1.06 (1.03-1.09) |
|  |  | no MVPA | 312,172 | 10,002 | 2,183,919.3 | 4.58 | 1.14 (1.11-1.18) |
|  | Moderate/heavy | MVPA | 241,472 | 5,435 | 1,702,439.7 | 3.19 | 1.22 (1.18-1.26) |
|  |  | no MVPA | 192,734 | 5,210 | 1,345,348.5 | 3.87 | 1.33 (1.29-1.38) |

*Incidence rates were calculated per 1,000 patient-years from the baseline population

**HR is adjusted for age, sex, income level, body mass index, previous history of hypertension and hyperlipidemia, duration, and previous medication for diabetes mellitus.

AF, atrial fibrillation; CI, confidence interval; DM, diabetes mellitus; HR, hazard ratio; IR, incidence rate, MVPA, moderate-to-vigorous physical activity

**Supplementary Table 10.** Definitions of comorbidities and outcomes

|  | ICD-10 codes with additional definitions | | Health examinations results |
| --- | --- | --- | --- |
| Comorbidities |  |  |  |
| Hypertension | I10-I13, I15 | Admission ≥1 or outpatient clinic ≥2, and prescription of anti-hypertensive drug (thiazide, loop diuretics, aldosterone antagonist, alpha-/beta-blocker, calcium-channel blocker, angiotensin-converting enzyme inhibitor, angiotensin II receptor blocker) | Systolic blood pressure ≥140 mmHg or diastolic blood pressure ≥90 mmHg |
| Dyslipidemia | E78 | Admission or outpatient department≥1, and prescription of lipid-lowering medication (statin, ezetimibe, fenofibrate) | Fasting total cholesterol ≥240 mg/dL |
| Chronic kidney disease |  |  | Creatinine clearance <60 mL/min |
| Socioeconomic status | |  |  |
| Low income |  | Composite of the lowest quartile of yearly income in addition to medicare beneficiaries |  |
| Outcomes |  |  |  |
| Atrial fibrillation | I48 | Admission ≥1 or outpatient clinic ≥2 |  |

ICD, international classification of diseases
